# Effect of Yi Gong San Decoction on Iron Homeostasis in a Mouse Model of Acute Inflammation

**DOI:** 10.1155/2016/2696480

**Published:** 2016-04-07

**Authors:** Qin Zheng, Yu Guan, Lemin Xia, Zhicheng Wang, Yiling Jiang, Xiaofeng Zhang, Jianying Wang, Guohua Wang, Yiqiong Pu, Jing Xia, Meihong Luo

**Affiliations:** ^1^Department of Hematology, Shanghai Baoshan Hospital of Integrated Traditional Chinese and Western Medicine (Baoshan Branch of Shuguang Hospital Affiliated to Shanghai University of Traditional Chinese Medicine), Shanghai 201999, China; ^2^Department of Central Laboratory Medicine, Shanghai Municipal Hospital of Traditional Chinese Medicine, Shanghai University of Traditional Chinese Medicine, Shanghai 200071, China; ^3^Department of Laboratory Medicine, Huashan Hospital, Shanghai Medical College, Fudan University, Shanghai 200040, China; ^4^College of Pharmacy, Shanghai University of Traditional Chinese Medicine, Shanghai 201203, China

## Abstract

We investigated the effect of Yi Gong San (YGS) decoction on iron homeostasis and the possible underlying mechanisms in a mouse model of acute inflammation in this study. Our findings suggest that YGS regulates iron homeostasis by downregulating the level of HAMP mRNA, which may depend on regulation of the IL-6/STAT3 or BMP/HJV/SMAD pathway during acute inflammation.

## 1. Introduction

Iron plays a pivotal role in cell survival and proliferation. It is thus an important source of nutrition in the competition between microbial pathogens and their hosts [1]. In humans, host defense responses to infectious agents modulate local and systemic iron availability, which disrupts infections such as malaria and tuberculosis [2]. Increased production of inflammatory cytokines can directly induce changes in iron homeostasis, which are characterized by reduction of both iron absorption and macrophage iron release. Hepatic bactericidal protein (hepcidin) provides a first line of defense at mucosal barriers, although it is less potent than many other antimicrobial peptides. Hepcidin impairs iron absorption and macrophage iron release and acts as a major hormonal regulator of iron homeostasis [1, 3]. The bone morphogenic protein (BMP)/hemojuvelin (HJV)/SMAD pathway is the major regulator of hepcidin expression that responds to iron status. Additionally, inflammation stimulates hepcidin via the interleukin- (IL-) 6/STAT3 pathway with support by activation of the BMP/HJV/SMAD pathway [4]. The expression of hepcidin in isolated primary hepatocytes increases in response to infection/inflammation stimulated by IL-6, IL-1, and LPS [5]. LPS is a component of the outer membrane of Gram-negative bacteria and elicits a potent inflammatory response when administered intravenously or intraperitoneally [6]. LPS induces hepcidin and causes hypoferraemia within hours of administration in both humans [7] and mice [3, 8]. Therefore, we investigated iron homeostasis in a mouse model of LPS-induced acute inflammation.

YGS originated from* Pediatric Medicine Card Straight Strategics*, a classic book of traditional Chinese medicine (TCM) that was written approximately 900 years ago. It has the functions of tonifying splenic Qi and gasification stagnation. YGS is based on Si Jun Zi decoction (SJZD) combined with Citri Unshius Pericarpium. SJZD can decrease serum levels of IL-6, CRP, and TNF. Early application of SJZD during enteral nutritional therapy can enhance the immune function of patients with gastrointestinal tumours [9]. In addition, YGS has been traditionally used in Korea to treat a variety of inflammatory diseases; pretreatment with YGS inhibited TNF-*α* and IL-6 production by LPS-stimulated mouse peritoneal macrophages [10]. Thus, YGS may maintain iron homeostasis by regulating the production of inflammatory cytokines. This study aimed to determine the effect of YGS on iron homeostasis and elucidate the underlying mechanisms to facilitate its clinical application.

## 2. Materials and Methods

### 2.1. Animals

Six-week-old C57BL/6 female mice were purchased from SLRC Laboratory Animals (Shanghai, China). The mice were housed in an environmentally controlled animal care facility and were used for experiments after 4 days of acclimation. Experiments were carried out according to the China Council on Animal Care guidelines after approval by the Institutional Animal Care Committee of Shanghai University of Traditional Chinese Medicine, Shanghai, China.

### 2.2. Preparation of YGS

YGS is composed of five different herbs: Ginseng Radix et Rhizoma (Ren-Shen), Glycyrrhizae Radix et Rhizoma (Gan-Cao), Citri Reticulatae Pericarpium (Chen-Pi), Atractylodis Macrocephalae Rhizoma (Bai-Zhu), and Poria (Fu-Ling). All of the five herbs were provided by our hospital pharmacy. After being soaked together for 1 h, the five herbs in equal dose were water decocted for 20 min, concentrated by distillation, dried through 60°C vacuum decompression, and homogenised into 100-mesh powders. 1 g dry powder was equal to 4.1 g herb (performed at the College of Pharmacy, Shanghai University of Traditional Chinese Medicine, Shanghai, China). The dosage given to mice was 10.57 g/kg which was equally effective to clinical routine dosage (each herb 15 g, total 75 g), calculated according to the surface area formula. The powder was dissolved in double-distilled water to a final concentration of 1.057 g/mL.

### 2.3. Multicomponent Analysis of YGS Powder

#### 2.3.1. Sample Preparation

0.3 g of accurately weighed fine powder was placed in a 50 mL centrifuge tube and ultrasonically extracted with 25 mL of 50% methanol (v/v) for 20 min. After centrifugation at 14000 rpm for 10 min, the supernatant was obtained and used as the test solution.

#### 2.3.2. UPLC/QTOF MS Conditions

Chromatographic separation was performed on Waters ACQUITY I-Class UPLC (Waters, Milford, MA, USA) equipped with a binary solvent manager, a sample manager, and a column manager. A Waters HSS T3 column (2.1 × 100 mm, 1.7 *μ*m) together with a Waters on-line filtrate 35°C was used. The mobile phase consisted of acetonitrile (B) and water containing 0.1% formic acid (v/v) (A) following a gradient elution program: 0–2 min: 15%–25% (B); 2–18 min: 25%–47% (B); 18–18.5 min: 47%–75% (B); 18.5–20 min: 75%–90% (B); 20–22 min: 90% (B); 22–22.1 min: 90%–15% (B); 22.1–26 min : 15% (B). The flow rate was set at 0.4 mL/min. 2 *μ*L of the test solution was injected for UPLC analysis.

High-accuracy mass spectrometric data were recorded on a Waters Xevo G2-S QTOF mass spectrometer (Waters, Manchester, UK). Tune parameters were set for MS^E^ experiments: capillary voltage, 2.5 kV (negative mode) and 2.0 KV (positive mode); sampling cone, 60 V; source offset voltage, 60 V; source temperature, 120°C; desolvation temperature, 450°C (negative mode) and 350°C (positive mode); cone gas flow, 30 L/h (negative mode) and 20 L/h (positive mode); desolvation gas, 900 L/h (negative mode) and 800 L/h (positive mode). The mass analyzer scanned over a mass range of 100–1500 Da within 0.1 s under a low collision energy at 6 V. High collision energy ramp of 20–90 V for negative mode and 40–90 V for positive mode was employed. Data calibration was performed using an external reference (LockSpray*™*) constant infused at 1 ng/*μ*L of leucine enkephalin (LE; Sigma-Aldrich, St. Louis, MO, USA) at a flow rate of 5 *μ*L/min, and with reference to the ion* m/z* 554.2615. Data acquisition was controlled by MassLynx V4.1 software (Waters Corporation, Milford, USA). Automatic metabolites characterization was performed using UNIFI 1.8 (Waters, Milford, USA) by the search of the TCM library.

### 2.4. Detection of Total Iron Concentration in YGS Powder

Total iron concentration in YGS Powder was determined by using an Agilent Technologies 7700 Series ICP-MS system equipped with ASX-500 Series ICP-MS Autosampler. The procedure was performed as previously described [11].

### 2.5. Animal Model

To induce acute inflammation, mice were injected with LPS (*Escherichia coli* serotype O127:B8, 1.5 mg/kg intraperitoneally; Sigma-Aldrich, Inc., USA) and sacrificed at 3, 6, 9, and 12 h thereafter. A CTL group was also established. All animals were given an equivalent volume of double-distilled water via intragastric administration for 7 successive days before being injected with LPS. To evaluate the effect of YGS on iron homeostasis* in vivo* and the underlying mechanisms, YGS (10.57 g/kg) was given to mice via intragastric administration for 7 successive days. Mice were injected with LPS on the following day and sacrificed at 3, 6, 9, and 12 h. A YGS control group was also established.

### 2.6. Specimen Collection

Mice were immediately killed with isoflurane, and blood was collected into serum separator tubes through cardiac puncture. The abdomen was then opened and liver samples were taken for tissue iron determination and ribonucleic acid (RNA) and protein isolation. Samples were stored at −80°C for later analysis.

### 2.7. Determination of Liver Iron

Liver tissue was processed as follows. About 100 to 200 mg of liver tissue was accurately weighed in a 1.5 mL centrifuge tube. Following the addition of 1 mL nitric acid solution, each centrifuge tube was vortex mixed after blending into the microwave digestion instrument resolution organisation and made up to 1.5 mL using nitric acid solution. Samples were then centrifuged at room temperature at 12,000 rpm for 3 min and then transferred to 96-well plates. After incubation for 10 min at room temperature, an ultraviolet spectrophotometer was used to determine the OD at 535 nm of the samples. Iron content was calculated using the following formula: iron concentration (*μ*g/g wet weight) = [(*A*_*t*_ − *A*_*b*_)*∗*(*n* + 0.75*W*)*∗*Fes (1 + *V*_*e*_)]/[(*A*_*s*_ − *A*_*b*_)*∗W∗V*_*e*_ × 1.1] (*A*_*T*_ = sample absorbance; *A*_*b*_ = absorbance blank hole; *A*_*s*_ = standard absorbance; Fes = standard iron content (*μ*g); *W* = wet weight; *n* = join tissue samples of acid volume (mL); *V*_*e*_ = acid extraction volume (mL)).

### 2.8. Determination of Serum Iron Levels

About 200 *μ*L of whole blood from the posterior orbital venous plexus was placed in serum separation tubes and centrifuged at 3000 rpm for 25 min at 4°C to separate the serum; approximately 40 *μ*L of serum was produced from each sample. In accordance with the manufacturer's instructions, 50 *μ*L of liquid iron buffer was added to the wells of a 96-well plate, as were 10 *μ*L aliquots of the standard and the test samples. A trace ultraviolet spectrophotometer A1 value wavelength (560 nm) was used. We added 1 *μ*L of iron chromogenic agent in each hole, 37°C, 10 min, using trace ultraviolet spectrophotometer A2 value wavelength (560 nm). Serum iron (*μ*g/dL) was calculated using the following formula: 500 × (A2a − A1a)/(A2b − A1b) (A2a, A2 specimens; A1a, A1 specimens; A2b, A2 standard samples; A1b, A1 standard samples). Serum and liver iron levels were measured by colourimetric assay (MI 48188, Pointe Scientific Inc., USA) using a NanoDrop2000 Spectrophotometer (Thermo Scientific Inc., USA).

### 2.9. IL-6 Assay

Serum IL-6 levels were determined using an ELISA kit according to the manufacturer's instructions (mouse IL-6 ELISA kit, M6000B; R&D Systems, Inc., USA). A standard dilution series of 3.12, 6.25, 12.5, 25, 50, and 100 pg/L was created. To each well of a 96-well plate was added 100 *μ*L of standard diluent RDIW together with 100 *μ*L of standard or sample. Following incubation at room temperature for 2 h, liquid was aspirated and wells were washed four times. Next, 200 *μ*L of IL-6 polymer was added, followed by incubation at room temperature for 2 h. Liquid was aspirated and wells were washed four times. Next, 200 *μ*L of substrate solution was added to each well, followed by incubation in the dark at a room temperature for 20 min. Next, 50 *μ*L of liquid was added to each well, and the plates were incubated at room temperature for 30 min. IL-6 levels were then determined using an ultraviolet spectrophotometer to read OD values (450 nm wavelength, calibration wavelengths of 540 and 570 nm). The standard curve was used to calculate IL-6 levels.

### 2.10. Determination of mRNA Levels

Total RNA was isolated using RNAiso Plus (D9108A, TaKaRa Bio Inc., Japan) and reverse-transcribed by PrimeScript reverse transcriptase using a real-time polymerase chain reaction (RT-PCR) system (TaKaRa Bio Inc., Japan). *β*-actin, HAMP, IL-6, BMP6, and HJV mRNA levels were measured using CFX96 RT-PCR Detection System (Bio-Rad, Inc., USA) with SYBR Premix Ex Taq Kit (DRR420A, Bio-Rad, Inc., USA). Expression levels were normalised to that of the housekeeping gene *β*-actin. The primers used are shown in Table 1.

### 2.11. Sodium Dodecyl Sulfate- (SDS-) Polyacrylamide Gel Electrophoresis and Western Blot Analysis

Livers were removed, rinsed in ice-cold phosphate-buffered saline, and used to prepare total protein extracts with RIPA Lysis Buffer (Beyotime Institute of Biotechnology, China) plus 1 mM phenylmethanesulfonyl fluoride and 0.1 to 2.0 mM sodium orthovanadate (Beyotime Institute of Biotechnology, China). Total protein extracts were separated in a 10% SDS-polyacrylamide gel and blotted onto nitrocellulose membranes (Bio-Rad, Inc., USA). The membranes were immunoblotted with antibodies to the following: phospho-STAT3, STAT3, *β*-actin, phospho-SMAD1/5/8, SMAD5 (Cell Signaling Technology, Inc., USA), and HJV (R&D Systems, Inc., USA). Anti-rabbit IgG, anti-mouse IgG, and anti-goat IgG (Cell Signaling Technology, Inc., USA) were used as secondary antibodies. Antigen-antibody complexes were visualised using an Immune-Star*™* Western C*™* Kit (Bio-Rad, Inc., USA) and analyzed using Image Lab*™* software (Bio-Rad, Inc., USA).

### 2.12. Statistical Analysis

All statistical analyses were performed using SPSS 22.0 software (IBM Inc., USA). Multiple comparisons were performed by one-way analysis of variance (ANOVA) followed by the Bonferroni correction. If data could not be compared using an equal variance *t*-test, a Kruskal-Wallis test and one-way ANOVA were used, followed by Student-Newman-Keuls or the Dunn* post hoc* test. Correlation coefficients were determined using Spearman's rank-order correlation method.

## 3. Results

### 3.1. Multicomponent of YGS Powder

**Figure 1:**
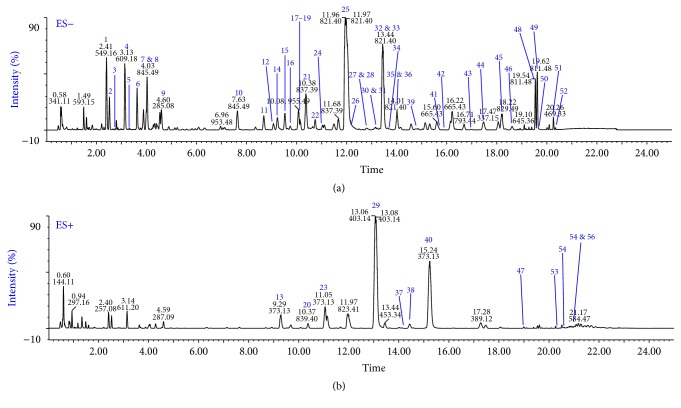
Base peak chromatograms of YGSP in negative mode (a) and positive mode (b).

By optimizing the gradient elution program, satisfactory separation of major peaks was achieved in both negative and position ion modes, as shown in Figure 1. The obtained MS^E^ data were further imported into UNIFI software for automatic components characterization. By comparison with the TCM library, 56 peaks were identified or tentatively characterized by element composition and fragment matching analyses (Table 2). Among them, 24 saponins should be from Glycyrrhizae Radix et Rhizoma, 19 saponins from Ginseng Radix et Rhizoma, 5 compounds from Citri Reticulatae Pericarpium, 5 from Poria, and 3 from Atractylodis Macrocephalae Rhizoma.

In contrast, compounds** 7**,** 8**,** 15**,** 25**,** 32**, and** 35**, from Glycyrrhizae Radix et Rhizoma or Ginseng Radix et Rhizoma, gave strong ion response in the negative ion mode, whilst the protonated precursors of compounds** 23**,** 29**, and** 40**, from Citri Reticulatae Pericarpium, were strong in positive ion mode. These compounds with strong responses as well as abundant secondary product ions lead to credible identification results. However, the compounds characteristic of Poria and Atractylodis Macrocephalae Rhizoma generated rather weak signals of precursor ions, without MS/MS fragments.

### 3.2. The Iron Element Level in YGS Decoction Could Not Directly Affect the Results

The iron concentration in YGS Powder was 2.56 mg per 100 g lyophilized powder weight. The dosage of YGS decoction given to the mice was 10.57 g/kg. The weight of the mice was from 18 g to 20 g. So, the iron taken by the mice from YGS decoction was not over 0.0015 mg. Mice were fed a standard iron rodent laboratory diet (232 mg iron/kg). Thus, precious little iron in YGS decoction could not directly affect the results.

### 3.3. YGS Increased Serum Iron by Decreasing Liver Iron Retention

**Figure 2:**
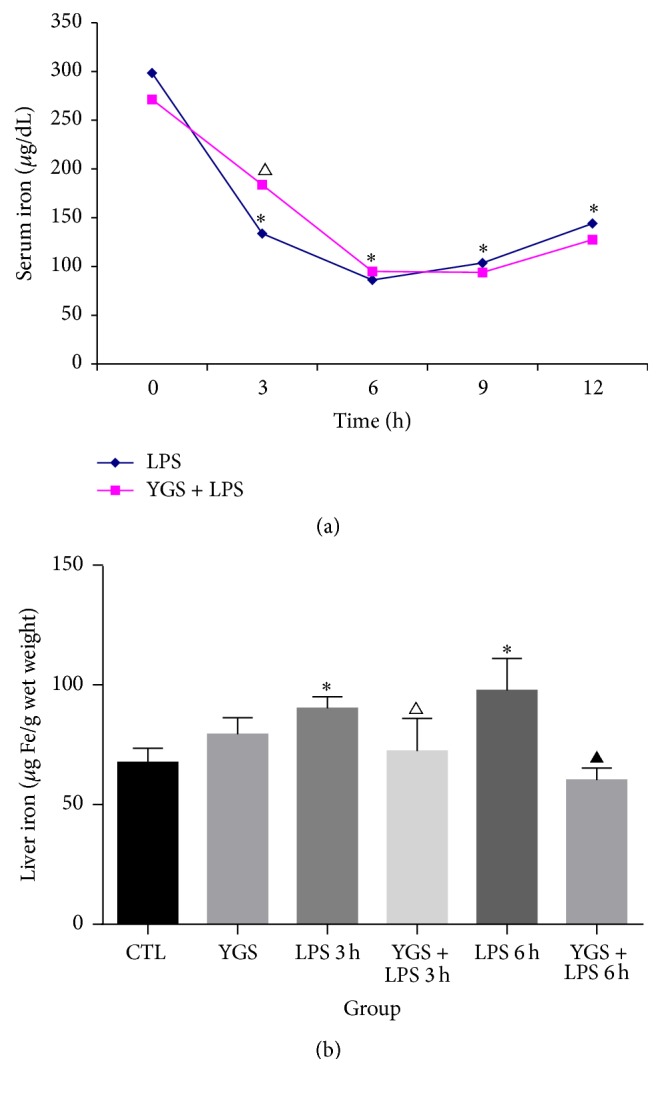
Effects of YGS on LPS-induced imbalanced iron homeostasis. (a) Colourimetric analysis of serum iron levels at 3, 6, 9, and 12 h after LPS administration. (b) Colourimetric analysis of liver iron levels at 3 and 6 h after LPS administration. This experiment was repeated twice, and the results are shown as mean plus or minus SD; *n* = 6. ^*∗*^ *P* < 0.05 versus the control group; ^△^ *P* < 0.05 versus the LPS 3 h group; ^▲^ *P* < 0.05 versus the LPS 6 h group.

In light of the influence of YGS on imbalanced iron homeostasis mediated by LPS, serum and liver iron concentrations were determined following preventive intervention with YGS. YGS alone had no significant effect on serum or liver iron levels. However, when compared with the LPS-treated control, YGS pretreatment significantly reduced the ability of LPS to decrease the serum iron level (Figure 2(a)) and iron retention in the liver (Figure 2(b)) at both 3 and 6 h after LPS injection (*P* < 0.05). Therefore, 3 and 6 h were used in subsequent analyses.

### 3.4. YGS Blockade of LPS-Mediated Hepcidin Induction via the IL-6/STAT3 Signaling Pathway

**Figure 3:**
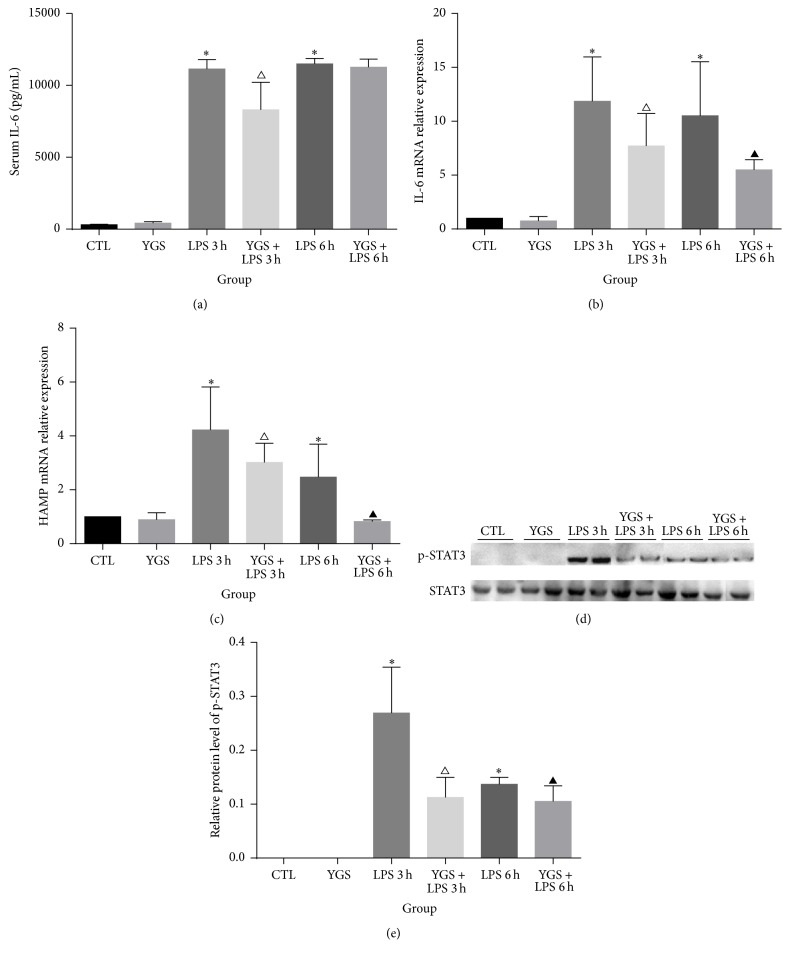
Levels of serum and liver IL-6 protein and liver HAMP mRNA and p-STAT3 protein. (a) ELISA of serum IL-6 levels. (b) Real-time PCR analysis of liver IL-6 and HAMP mRNA levels. (c) Western blotting analysis of p-STAT3 and STAT3 protein levels. (d, e) Western blotting analysis of p-STAT3/STAT3 protein levels expressed as densitometry values. This experiment was repeated twice, and the results are shown as mean plus or minus SD; *n* = 6. ^*∗*^ *P* < 0.05 versus the control group; ^△^ *P* < 0.05 versus the LPS 3 h group; ^▲^ *P* < 0.05 versus the LPS 6 h group.

LPS injection leads to the production of the inflammatory cytokine IL-6 [12], which has been identified as a major inducer of hepcidin through the IL-6/STAT pathway [3]. Inhibiting LPS-induced secretion of IL-6 decreases hepcidin levels. Hence, in this study, a mouse model was used to evaluate the effect of YGS on LPS-induced IL-6 release. Compared with the LPS-treated control, YGS pretreatment significantly reduced the ability of LPS to increase serum IL-6 levels (Figure 3(a)) at 3 h, as well as IL-6 mRNA expression (Figure 3(b)) at both 3 and 6 h (*P* < 0.05). Hence, YGS blocked IL-6 increase after LPS injection* in vivo*. LPS-mediated HAMP mRNA expression (Figure 3(c)) was also significantly decreased by YGS at both 3 and 6 h (*P* < 0.05), although YGS alone did not significantly reduce HAMP mRNA levels. YGS inhibited STAT3 phosphorylation only at 3 h (*P* < 0.05) (Figures 3(d) and 3(e)).

### 3.5. BMP/HJV/SMAD Signaling Pathway May Contribute to Maintenance of Iron Homeostasis by YGS

**Figure 4:**
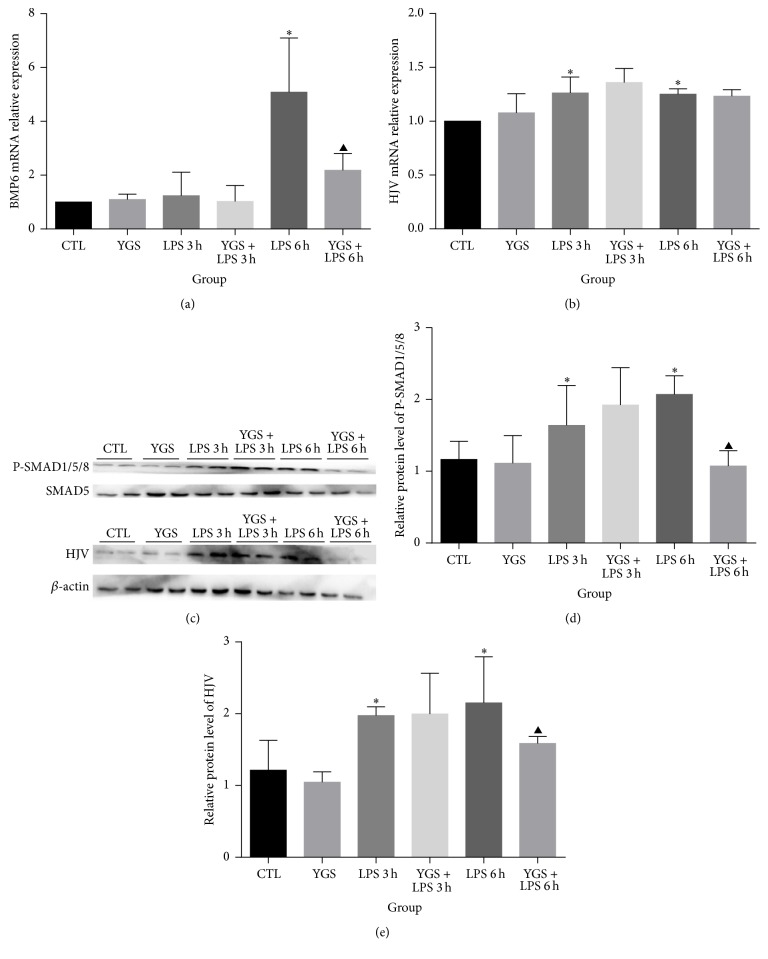
Hepatic levels of BMP6 and HJV mRNA and p-SMAD1/5/8 and HJV protein. Real-time PCR analysis of liver BMP6 (a) and HJV (b) mRNA levels at 3 and 6 h after LPS administration. Western blotting analysis of p-SMAD1/5/8 (c, d) and HJV (c, e) protein levels expressed as densitometry values. This experiment was repeated twice, and the results are shown as means plus or minus SD; *n* = 6. ^*∗*^ *P* < 0.05 versus the control group; and ^▲^ *P* < 0.05 versus the LPS 6 h group.

There is a crosslink between the iron and cytokine-dependent pathways of hepcidin upregulation. The integrity of the BMP/HJV/SMAD pathway is required to activate hepcidin [13]. Hence, we determined whether YGS downregulates hepcidin through BMP/HJV/SMAD pathway. Compared with the CTL group, LPS resulted in a significant increase in the liver HJV mRNA level (Figure 4(b)) and P-SMAD1/5/8 (Figures 4(c) and 4(d)) and HJV (Figures 4(c) and 4(e)) protein levels at both 3 and 6 h, and BMP6 mRNA expression (Figure 4(a)) only at 6 h (*P* < 0.05). Moreover, YGS pretreatment significantly inhibited the increase in the BMP mRNA level (Figure 4(a)) and P-SMAD1/5/8 (Figures 4(c) and 4(d)) and HJV (Figures 4(c) and 4(e)) protein levels only at 6 h (*P* < 0.05).

## 4. Discussion

Metabolic iron homeostasis is regulated by several factors but is most closely related to hepcidin, the central mediator of iron homeostasis. As it binds to its target iron-export protein, ferroportin (Fpn), hepcidin stimulates internalisation and degradation of Fpn and reduces the quantity of Fpn on the small intestinal mucosa and in macrophages [14]. This process serves to control dietary iron absorption, iron release from storage sites, and iron bioavailability in the body, therefore regulating the balance among iron absorption, iron utilisation, and iron storage [15].

In inflammatory conditions, hepcidin is augmented by increased levels of the inflammatory factor IL-6. Upon binding to its membrane-bound receptor glycoprotein (gp)80 on hepatocytes, IL-6 further interacts with the gp130 membrane glycoprotein to induce STAT3 (signal transducer and activator of transcription) phosphorylation [12]. Phosphorylated STAT3 enters the nucleus and upregulates transcription of the gene encoding hepcidin. This increases the circulating hepcidin level, followed by enhanced Fpn internalisation and degradation [1]. As a result, dietary iron absorption and iron release from storage sites are restricted.

The spleen is a larger concept in TCM than in Western medicine. According to TCM theory, the spleen has the central function of transporting and transforming nutrient substances, and this function plays a major role in erythropoiesis. Hence, it is plausible that iron absorption, transport, and transformation are controlled by the spleen. Furthermore, we hypothesised that an imbalance of iron homeostasis might be improved by promoting movement of splenic Qi [16]. YGS, a representative TCM prescription for promoting the movement of splenic Qi, was investigated because it has been reported to regulate the expression of inflammatory factors.

We first investigated the effect of YGS on LPS-induced imbalanced iron homeostasis in mice. Injection of 1.5 mg/kg of LPS into mice resulted in serum iron deficiency and liver iron retention within 3, 6, 9, or 12 h. Both the minimum serum iron level and maximum liver iron level occurred at 6 h. Serum and liver iron levels differed significantly between groups with and without YGS pretreatment before LPS injection, particularly at 3 and 6 h. Thus, YGS pretreatment blocked the ability of LPS to decrease serum iron levels and increase liver iron levels. This finding suggests that YGS can adjust the abnormal iron distribution under inflammatory conditions.

We next investigated the mechanisms underlying the maintenance of iron homeostasis by YGS. LPS injection leads to the production of the inflammatory cytokine IL-6, which is a major hepcidin inducer [12]. Inhibition of LPS-induced secretion of IL-6 decreases hepcidin levels. IL-6 directly regulates hepcidin through induction and subsequent promoter binding of STAT3, which is necessary for IL-6-mediated hepcidin induction [17]. Hence, we first investigated the STAT3 pathway. We observed the downregulation of IL-6 mRNA in mice pretreated with YGS prior to LPS administration at both 3 and 6 h. However, serum IL-6 was downregulated only at 3 h. The kinetics of mRNA and protein levels differed, likely because serum IL-6 protein has a longer half-life than IL-6 mRNA [18]. In addition, the use of incremental doses or prolonged delivery of YGS may result in more persistent downregulation of serum IL-6 levels; this warrants further research.

As expected, HAMP mRNA levels at 3 h were downregulated due to the decreased IL-6 mRNA and protein levels. p-STAT3 levels also decreased at 3 h, which indicates that YGS may maintain iron homeostasis by regulating IL-6/STAT3/hepcidin pathway. However, HAMP mRNA levels at 6 h were also downregulated by YGS pretreatment, although serum IL-6 and p-STAT3 levels were unaffected. Therefore, YGS may have inhibited inflammation caused by hepcidin production via another signaling pathway.

The BMP signaling pathway is involved in regulating hepcidin expression in the liver. BMPs are potent inducers of hepcidin production. The interactions of BMPs with BMP receptors result in the phosphorylation of a subset of SMAD proteins (SMAD1/5/8) and subsequent formation of a heteromeric complex with SMAD4, which translocates to the nucleus and induces the transcription of target genes [19]. HJV, a member of the repulsive guidance molecule (RGM) family, acts as a BMP coreceptor and triggers the binding of BMP ligands to BMP receptors to enhance hepcidin expression. Regulation of BMP/HJV/SMAD signaling occurs also after the phosphorylation of SMAD1/5/8, which ensures fine-tuning at the cytosolic level. STAT3-inducible hepcidin expression is also influenced by BMP-dependent SMAD activation. The BMP/HJV/SMAD signaling pathway modulates IL-6-inducible STAT3 pathway [12]. Thus, it may cooperate and enhance IL-6-dependent stimulation and may represent a connecting component between iron status and inflammation.

As mentioned earlier, we further explored BMP/HJV/SMAD pathway. BMP6 mRNA and P-SMAD1/5/8 and HJV protein levels were downregulated at 6 h by YGS pretreatment, which was correlated with the HAMP mRNA levels. Thus, we speculated that regulation of the BMP/HJV/SMAD pathway by YGS may contribute to the downregulation of hepcidin, resulting in the restoration of iron homeostasis. Our findings show that YGS regulates iron homeostasis by downregulating HAMP mRNA, which may depend on the regulation of the IL-6/STAT3 or BMP/HJV/SMAD pathway during acute inflammation.

Anaemia of chronic disease (ACD) is the most common anaemia secondary to various chronic infections, chronic inflammation, and malignancies. Increased production of inflammatory cytokines can directly induce changes in iron homeostasis, which are characterized by the reduction of both iron absorption and macrophage iron release, resulting in ACD [20, 21]. In contrast to iron deficiency anaemia, hypoferraemia in ACD is not due to iron deficiency; instead, the impaired iron absorption and utilisation regulatory mechanisms result in imbalanced iron homeostasis [22]. The changes of iron metabolism in the mice caused by LPS injection through hepcidin-induced inflammation pathway, which is one of the important ACD pathogenesis, imitate ACD pathological state although the mice can not be anaemic [6, 23]. So, if YGS decoction could improve this mimic ACD pathological state, it is possible to be used to prevent or treat ACD. Further studies on the YGS decoction are maybe important in science and application.

## Figures and Tables

**Table 1:** PCR primers, product sizes, and annealing temperatures.

Gene	PCR primer sequence	Temperature (°C)
*β*-actin	5′-AGCTGAGAGGAAATCGTGCG-3′	59.8
	5′-GTGCCACCAGACAGCACTGTG-3′	
HAMP	5′-AGCACCACCTATCTCCATCAAC-3′	57.0
	5′-TGTCTCTCTTCCTTCTCTTCTGC-3′	
IL-6	5′-GGAGAGGAGACTTCACAGAGGA-3′	57.0
	5′-ATTTCCACGATTTCCCAGAGA-3′	
BMP6	5′-CAGGAGCATCAGCACAGAGA-3′	59.8
	5′-GTCACCACCCACAGATTGC-3′	
HJV	5′-TGCTAACCTTGGGAGTCACG-3′	59.8
	5′-TCCTCTGCTACCCTGATGGA-3′	

**Table 2:** Information of 56 compounds identified from YGNP by UPLC/QTOF MS.

Number	Retention time (min)	Identification	TCM
1	2.42	Licurazide or its isomer	GC
2	2.52	Isoliquiritin or its isomer	GC
3	2.75	Ferulic acid	GC
4	3.14	Hesperidin or neohesperidin	CP
5	3.32	20-*O*-Glucopyranosyl ginsenoside Rf	RS
6	3.63	Licurazide or its isomer	GC
7	4.01	Ginsenoside Re	RS
8	4.03	Ginsenoside Rg1	RS
9	4.60	Licochalcone B	GC
10	7.64	Ginsenoside Rf	RS
11	8.74	Uralsaponin U/N or licorice saponin G2	GC
12	9.08	Ginsenoside 20(*S*)-Rg2	RS
13	9.28	Sinensetin or its isomer	CP
14	9.31	Ginsenoside Ra1 or Ra2	RS
15	9.53	Ginsenoside Rb1	RS
16	9.81	Ginsenoside F1	RS
17	10.13	Ginsenoside Ro	RS
18	10.14	Ginsenoside Ra2	RS
19	10.15	Ginsenoside Rb2, Rb3, or Rc	RS
20	10.37	Glabrolide or isoglabrolide	GC
21	10.42	Uralsaponin U/N or licorice saponin G2	GC
22	10.74	Ginsenoside Rb2, Rb3, or Rc	RS
23	11.05	Sinensetin or its isomer	CP
24	11.08	Uralsaponin U/N or licorice saponin G2	GC
25	11.95	Glycyrrhizic acid or its isomer	GC
26	12.08	Ginsenoside Rd	RS
27	12.80	Glycyrrhizic acid or its isomer	GC
28	12.82	Licorice saponin K2 or its isomer	GC
29	13.07	Nobiletin	CP
30	13.17	Ginsenoside Ro	RS
31	13.22	Licoflavone A or its isomer	GC
32	13.44	Glycyrrhizic acid or its isomer	GC
33	13.48	Licorice saponin K2 or its isomer	GC
34	13.64	Licobenzofuran	GC
35	14.01	Glycyrrhizic acid or its isomer	GC
36	14.04	Licorice saponin K2 or its isomer	GC
37	14.11	Atractylenolide I or its isomer	BZ
38	14.61	Uralsaponin C/P or licorice saponin J2	GC
39	14.75	Ginsenoside Rg3 or its isomer	RS
40	15.23	Sinensetin or its isomer	CP
41	15.68	Uralsaponin V/W or licorice saponin C2	GC
42	15.89	Poricoic B or its isomer	FL
43	16.99	Licoflavone A or its isomer	GC
44	17.47	Licoflavone A or its isomer	GC
45	18.20	Ginsenoside 20(S)-Rg3	RS
46	18.61	Ginsenoside 20(R)-Rg3	RS
47	18.99	3*β*-Hydroxyatractylon or atractylenolide II	BZ
48	19.54	Ginsenoside Rg4 or Rg6	RS
49	19.62	Ginsenoside Rg4 or Rg6	RS
50	19.71	Stractylenolide I or its isomer	BZ
51	20.27	Glycyrrhetinic acid or its isomer	GC
52	20.36	Glycyrrhetinic acid or its isomer	GC
53	20.44	Poricoic B or its isomer	FL
54	20.63	Dehydropachymic acid	FL
55	21.03	Pachymic acid	FL
56	21.09	Trametenolic acid or its isomer	FL

Note: GC (Gan-Cao): Glycyrrhizae Radix et Rhizoma; RS (Ren-Shen): Ginseng Radix et Rhizoma; CP (Chen-Pi): Citri Reticulatae Pericarpium; FL (Fu-Ling): Poria; BZ (Bai-Zhi): Atractylodis Macrocephalae Rhizoma.
